# The microbial removal of bisphenols in aquatic microcosms and associated alteration in bacterial community

**DOI:** 10.1007/s11356-023-28305-2

**Published:** 2023-06-29

**Authors:** Magdalena Noszczyńska, Magdalena Pacwa-Płociniczak, Kinga Bondarczuk, Zofia Piotrowska-Seget

**Affiliations:** 1 Institute of Biology, Biotechnology and Environmental Protection, Faculty of Natural Sciences, University of Silesia in Katowice, Jagiellońska 28, 40-032 Katowice, Poland; 2 Centre for Bioinformatics and Data Analysis, Medical University of Bialystok, Białystok, Poland

**Keywords:** Endocrine-disrupting chemicals, Bioremediation, Bisphenols, Bacterial consortium, Bioaugmentation, Autochtonous community

## Abstract

The concept of the study resulted from numerous concerns around bisphenol A (BPA) and bisphenol S (BPS) in aquatic environments. In this study, river water and sediment microcosms highly polluted with bisphenols and bioaugmented with two BPs-removing bacterial strains were constructed. The study aimed to determine the rate of high-concentrated BPA and BPS (BPs) removal from river water and sediment microniches, and the effect of water bioaugmentation with bacterial consortium on the removal rates of these pollutants. Moreover, the impact of introduced strains and exposure to BPs on the structural and functional composition of the autochthonous bacterial communities was elucidated. Our findings indicate that the removal activity of autochthonous bacteria was sufficient for effectively BPA elimination and reducing BPS content in the microcosms. The number of introduced bacterial cells decreased continuously until day 40, and on consecutive sampling days, no bioaugmented cells were detected. Sequencing analysis of the total 16S rRNA genes revealed that the community composition in bioaugmented microcosms amended with BPs differed significantly from those treated either with bacteria or BPs. A metagenomic analysis found an increase in the abundance of proteins responsible for xenobiotics removal in BPs-amended microcosms. This study provides new insights into the effects of bioaugmentation with a bacterial consortium on bacterial diversity and BPs removal in aquatic environments.

**Supplementary Information:**

The online version contains supplementary material available at 10.1007/s11356-023-28305-2.

## Introduction

Since the 1950s, bisphenol A (BPA) has been used extensively to manufacture polycarbonate plastic products such as food and beverage packaging, medical products, electronics, and epoxy resins. The first reports of its harmfulness to animals and humans appeared in the 1990s. Numerous studies have proved that BPA increases the risk of adverse health effects (Sharma et al. 2018). Due to its negative impact on living organisms, BPA has started to be replaced by its analog bisphenol S (BPS), in which the dimethyl methylene group (C(CH_3_)_2_) is substituted with a sulfone group (SO_2_). Such a chemical structure makes BPS resistant to removal (Fang et al. 2020). Moreover, recent studies have demonstrated that BPS, similarly to BPA, shows disruptive endocrine activity and is cancerogenic, teratogenic, and cytotoxic (Harnett et al. 2021). In addition, the worldwide growth in the production and consumption of these BPs has resulted in their detection in the aquatic environment (Noszczyńska and Piotrowska-Seget 2018).

Frequently, due to the incomplete removal of BPs in wastewater treatment plants (WWTPs) (Batukbhai et al. 2022), they may contaminate vital aquatic resources such as drinking water, surface water, and groundwater. In these resources, BPs concentrations range from a few nanograms to several milligrams per liter, which impairs the water quality (Bogunović et al. 2021). Ferrer-Polonio et al. (2021) suggested that in municipal WWTPs, BPA may reach concentrations higher than 5 mg L^−1^ due to discharges coming from industry and hospitals or especially when landfill leachates are transported to the plants. In turn, the maximum concentration of BPS was 7.2 μg L^−1^ in the Adyar River, India (Yamazaki et al. 2015). However, increasing the use of BPS will lead to higher environmental load (Ding et al. 2020). It is currently believed that high concentrations of BPs in water will result in their increased accumulation in aquatic biota (Czarny-Krzymińska et al. 2023). Although autochthonous bacterial communities may well degrade organic pollutants in water, little has been achieved regarding the effect of high BPs concentrations on the removal potential of these communities. Additionally, it should be taken into account that there are threshold concentrations of xenobiotics necessary for the induction of catabolic enzymes, below which no removal is observed. Moreover, differences in the kinetics of the same removal processes are often observed, but with different substrate concentrations and competition with other carbon sources at a low level (Zur et al. 2020). In various studies, the degradation of BPA in river water, sediment, and sludge from WWTPs was investigated (Kang and Kondo 2002; Zhao et al. 2008; Danzl et al. 2009; Mohapatra et al. 2011; Fang et al. 2020). Although various biodegradation pathways of this compound have been identified, the structure, effects, and fate of the intermediate metabolites still have to be explained (Im and Löffler 2016). Until now, two main BPA biodegradation mechanisms have been revealed. The first involves the oxidative skeletal rearrangement of an aliphatic methyl group in the BPA molecule, while the second involves hydroxylation of one or two phenolic rings followed by aromatic ring cleavage. Detailed pathways of biological decomposition of BPS is not known (Noszczyńska and Piotrowska-Seget 2018). Furthermore, since the alteration of microbial community structure is crucial for the ecosystem, it is important to characterize bacteria composition during removal of BPs present at high concentrations. These data are essential to design and develop sustainable in situ bioremediation technologies.

The effectiveness of BPs removal can be further improved by bioaugmentation. However, the bioaugmentation efficiency is highly dependent on the local surroundings, mainly due to the interplay between functional strains and the indigenous microbiome, resulting in strong structural shifts with neutral, beneficial, or detrimental impacts. The behavior of bioaugmented strains depends on their ability to survive and grow in a new environment, compete with indigenous microorganisms for nutrient acquisition, and resist changing environmental conditions such as pH, temperature, and water availability (Festa et al. 2016).

Here, we present the results of the first detailed investigation on the bioremediation potential of native river water and sediment microniches and the effect of augmenting water with a BPs-removing consortium on BPs removal rates. The consortium was made up of *Pseudomonas* sp. BG12 and *Acinetobacter* sp. K1MN, which could use BPA and BPS as sole carbon and energy sources for growth. The influence of bioaugmentation and exposure to BPs on the microbial community structure and changes in its functional dynamics was studied. The survivability of bacteria used for augmentation in the river water and sediment microcosms was monitored.

## Materials and methods

### Chemicals and sampling

BPA and BPS were obtained from Sigma-Aldrich (St. Luis, MO, USA). HPLC grade acetonitrile and ethanol were purchased from S. Witko - JT Baker (Lodz, Poland). Water used as a HPLC solvent was purified with a Direct-Q Water Purification System (Merck). All other chemicals were of analytical grade and purchased from Merck.

Water and sediments samples were collected from Rawa River in South Poland (50° 15′ 36″ N 19° 01′ 41″ E). The river is a sewage receiver from the wastewater treatment plant Klimzowiec in Chorzów, Poland. The water sample was collected under 10 cm of the water surface, while the sediment samples were taken from depths 0 to 10 cm. The samples were packed into sterile glass jars and immediately transferred to the laboratory and used to prepare microcosms.

### Bacterial strains and their labeling with a fluorescent protein

In this study, two bacterial strains showing the ability to BPs removal were used. These strains were isolated from two highly anthropogenically polluted areas. *Pseudomonas* sp. BG12 (GenBank MN061282, family: *Pseudomonadaceae*) was isolated from soil collected from Petrochemia-Blachownia SA, Kędzierzyn Koźle (Poland). The soil was added to the basal salt medium (BSM) (Badiefar et al. 2015) supplemented with BPA at a concentration of 1 mg L^−1^ and incubated at 28°C with rotary shaking (120 rpm). After 3 days, 1 mL of this culture was transferred into a fresh BSM medium with 10 mg L^−1^ of BPA and re-incubated. This step was repeated with progressively higher content of BPA (up to 100 mg L^−1^). *Acinetobacter* sp. K1MN (GenBank MN061280; family: *Moraxellaceae*) was isolated from Kalina pond, Świętochłowice (Poland). The water was mixed with BSM in a ratio of 1:9, supplemented with gradually increasing BPS concentration (1–100 mg L^−1^), and incubated under the same conditions as described above. The cultures were then plated on BSM with appropriate BPs (100 mg L^−1^). Morphologically distinct bacterial strains were tested for BPs removal abilities using high-performance liquid chromatography (HPLC). BG12 and K1MN strains showed the highest potential for removal of BPA (elimination of 60 mg L^−1^ in 15 days) and BPS (elimination of 20 mg L^−1^ in 15 days), respectively, and were chosen for further studies.

K1MN and BG12 were labeled with the tetracycline plasmids EGFP and mCherry, respectively, using triparental mating. The donor strains were *E. coli* DH5α carrying the EGFP-plasmid pMP4655 and *E. coli* DH5α carrying the mCherry-plasmid pMP7604, respectively. As a helper strain, *E. coli* H2013 was used. All strains were grown in Luria-Bertani (LB) medium, and the recipient strains were grown to reach OD_600_=0.7, whereas donor and helper strains were grown to reach OD_600_= 0.3–0.4. Additionally, the medium for the donor strain was supplemented with tetracycline at the final concentration of 20 μg mL^−1^. When the appropriate OD_600_ was reached, the bacterial strains were centrifuged (3000 rpm, 5 min, 22°C), and the pellets were re-suspended in 400 μL of fresh LB. 30 μL of each bacterial suspension was transferred onto a nitrocellulose filter in a Petri dish with LB medium and carefully mixed together. After 16 h of incubation at 30°C, fluorescent proteins (FP)-labeled recipient strains were isolated on the BSM medium supplemented with 100 mg L^−1^ appropriate BPs and tetracycline (20 μL mL^−1^). The stability of the labeling plasmid was confirmed by subculturing labeled K1MN, and BG12 on BSM supplemented with BPA or BPS (10 mg L^−1^) plates without the addition of tetracycline.

The fluorescence of the strains was checked using a Zeiss Axio Imager.Z.2 fluorescence microscope interfaced with the ZEN Pro software (Carl Zeiss).

### Experimental design

Microcosms were prepared in 1000-mL Erlenmeyer flasks with 500 mL of river water plus 50 g of wet sediment, 10 mg L^−1^ BPA, 10 mg L^−1^ BPS, and the FP-tagged bacterial consortium (Table 1). Before being added to microcosms, each BPs was first dissolved in 70% ethanol to receive the concentration of 10 mg mL^−1^ and filtered through a 0.22-μm Millipore filter. Microcosms were supplemented with nystatin (4 g L^−1^) and actidione (4 g L^−1^) to inhibit fungal growth. BSM or sterilized by autoclaving river water and sediment were used to prepare microcosms VIII and VII, respectively.

**Table 1:** Composition of created microcosms

Microcosms	Composition:
I.	water and sediment, (control)
II.	water and sediment, BPs, (control)
III.	consortium, water and sediment, (control);
IV.	consortium, water and sediment, BPs;
V.	consortium, water and sediment, BPS;
VI.	consortium, water and sediment, BPA;
VII.	consortium, autoclaved water and sediment, BPs, (control);
VIII.	consortium, BPs, (control).

K1MN and BG12 were used to prepare consortium. The strains were separately cultured in Luria-Bertani (LB) medium at 28°C in a horizontal shaker at 120 rpm. After 24 h, the culture was centrifuged (4700 rpm, 15 min, 4°C) and rinsed three times with sterile, deionized water, followed by re-centrifugation (4700 rpm, 15 min, 4°C). The 0.4 g of the wet weight of each bacterial strains (Xiong et al. 2017a) was then aseptically transferred to the appropriate microcosms.

Each type of microcosm treatment had three replicates for a total microcosm count of 24. All microcosms were incubated on a horizontal shaker at 120 rpm at 28°C for 70 days.

### Determination of BPs residual concentration

Every 10 days, samples from microcosms II, IV–VIII (containing 5 mL of BSM or river water + 1 g of sediment) were collected from each microcosm (Xiong et al. 2017b). The samples were extracted twice with 10 mL of 70% ethanol and sonicated for 60 min. The pellet was discarded. The supernatant was dried under a gentle stream of N_2_ in a water bath at 50°C and dissolved in 2 mL of 70% ethanol. Preliminary experiments showed that the extraction efficiency was 100 ± 1.64% for BPA and 100 ± 0.83% for BPS.

The extract was filtered with a 0.2-μm RC membrane filter (Hahnemuehle, Germany) and analyzed by HPLC (Shimadzu Kyoto, Japan) with a quaternary pump (model LC-20AD). Detection was performed by connecting in tandem two detectors: (i) fluorescent detector (Shimadzu, model RF-20AXS), set at the excitation wavelength of 273 nm and the emission wavelength of 305 nm for BPA analysis, and (b) UV detector (Shimadzu, model SPD-M20A) set at 220 nm for BPS analysis. Blank extracts (extraction of ultrapure water) served as controls. The chromatographic data were processed by the LabSolution software. Separation was achieved using the Phenomenex Synergi 4 μm Hydro-RP (150 × 4.6 mm) column, protected by an AQ C18 guard column (Phenomenex, Torrance CA, USA). BPA was eluted with a gradient of acetonitrile (A)–water (B) with a flow rate of 0.5 mL min^−1^. The injection volume was 15 μL. The gradient program was [time (min)/%A/%B]: 0/0/100, 5/50/50, 10.5/95/5, 15.5/50/50, 30/10/90. BPS was analyzed using a gradient program consisting of a mixture of A and B at a flow rate of 1 mL min^−1^. The injection volume was 15 μL. The gradient program was [time (min)/%A/%B]: 0/0/100, 25/90/10, 26/10/90, 35/10/90. Under these conditions, the retention times were 17.452 ± 0.04 min for BPA and 12.698 ± 0.08 min for BPS.

The calibration curve for BPA was obtained from a linear regression program by concentrations versus detector responses using concentration levels for six standards. These working solutions were prepared from stock solution of 10 μg mL^−1^ at concentrations of 0.3125; 0.625; 1.25; 2.5; 5; 10 μg mL^−1^. The correlation coefficient of peak height to concentration was 0.999.

The calibration curve for BPS was done using concentration levels for five standards: 0.5; 10; 50; 100; 150 μg mL^-1^. These standards were prepared from a stock solution of 50 mg mL^−1^. The correlation coefficient of peak height to concentration was 0.998.

The disappearance of BPA fitted into the second-order (1/*C* = *kt* + 1/*C*_0_), while BPS into the pseudo-zero order (*C= C*_*0*_ *- kt*) kinetic models. These algorithms, where *C* is a concentration of BPA/BPS (mM) at some time, *C*_*0*_ is an initial concentration (mM) of BPA/BPS, and *t* is time (day), were used to determine the removal rate (*k*) of BPs. The half-life (*t*_*1/2*_) of BPA and BPS removal was calculated using the equation *t*_*½*_ *=* 1/*kC*_0_*,* and *t*_*1/2*_ *= C*_0_/2*k*, respectively.

### Survival of FP-tagged bacterial strains in the microcosms

Once every 10 days, the survival of FP-tagged bacterial strains in microcosms IV, V, and VI was examined. Ten microliters from each microcosm were transferred into a Thoma chamber to count the K1MN and BG12 cells under Zeiss Axio Imager.Z.2 fluorescence microscope interfaced with the ZEN Pro software (Carl Zeiss). The total number of BG12 and K1MN was estimated based on the cells counted in at least 10 medium squares. A 3D response surface plot showing the correlation between bacteria counts, amount of BPs, and sampling time was made using SigmaPlot 14.0 software.

### DNA extraction and Illumina MiSeq sequencing

Total genomic DNA was extracted from microcosms I-III on the 1^st^ day, and from microcosms no. I–VI on the 35^th^, and 70^th^ day of the experiment. The genomic DNA of each water–sediment microcosm was extracted using DNeasy PowerWater and PowerSoil Kits (Qiagen) according to the manufacturer’s recommendations. The yield and purity of the DNA were determined using NanoPhotometer NP80 (Implen GmbH, München, Germany). The isolated DNA samples were stored at −20°C. The 16S rRNA libraries were constructed using bacterial primers 341F (5′-CCTACGGGNGGCWGCAG-3′) and 785R (5′-GACTACHVGGGTATCTAATCC-3′) for the V3–V4 region (Klindworth et al. 2013). Each library was prepared using the Q5 Hot Start High-Fidelity DNA Polymerase (NEBNext – New England BioLabs, Ipswich, MA, USA) according to the manufacturer’s instructions. Paired-end (PE, 2 × 250 nt) sequencing was performed on an Illumina MiSeq instrument (MiSeq Reagent kit v2) following the manufacturer’s protocols (Illumina, Inc., San Diego, CA, USA). The sequences generated in this study were deposited in the GenBank SRA database under BioProject accession PRJNA674995.

### Bioinformatic analysis

The automatic primary analysis and the demultiplexing of the raw reads were performed on MiSeq using the MiSeq Reporter (MSR) v2.4 software (BaseSpace). The sequences were subsequently processed on the CLC Genomics Workbench v20.0.4 (QIAGEN, Aarhus A/S, http://www.clcbio.com) as described by Pacwa-Płociniczak et al. (2020). Briefly, the adapter sequences were removed using the Trim Reads tool. Read trimming was performed using the default parameters (trim using quality scores= 0.05 and trim ambiguous nucleotides= 2). Samples with low coverage (low number of reads) were excluded from subsequent analysis. Paired reads were merged, and chimeric sequences were removed in the CLC microbial genomics module v1.1 using the default settings. Closed reference operational taxonomic unit (OTU) picking at 97% similarity was also performed. Greengenes v13_8 99% (DeSantis et al. 2006) for 16S rRNA (bacteria and archaea) was used as the reference OTU database. The alpha diversity parameters (OTU, Chao1-bias corrected, Shannon and Simpson indices) were generated using the CLC Microbial Genomics Module v20.1.1. Data was normalized using rarefaction method. Metagenomic biomarkers were identified and visualized by Linear Discriminant Analysis Effect Size (LEfSe) (Segata et al. 2011). The functional prediction was performed using PICRUSt2 with default options (Douglas et al. 2019).

### Statistical analysis

Statistical analysis was performed using STATISTICA 13.0 PL software (StatSoft, Tulsa, OK, USA). Statistically significant differences were accepted at a *p*-value of less than 0.05. Statistical significance between datasets was tested by a one-way analysis of variance (ANOVA) followed by a post hoc least significant difference (LSD) test.

For beta diversity, Bray-Curtis dissimilarity coefficients were calculated, and principal coordinate analysis (PCoA) was performed to visualize the beta diversity distance matrix. To describe significant changes in beta diversity of microbial communities dependent on treatment and time of the experiment, the PERMANOVA analysis tool was used with the number of permutations 99.999. Furthermore, to visualize dissimilarity among microbial communities from tested microcosms, a heat map and clustering analysis using the Euclidean distance and hierarchical clustering with the average linking method with filtering settings based on the relative abundance was performed.

## Results and discussion

### Removal of BPs from water-sediment microcosms

**Fig. 1:**
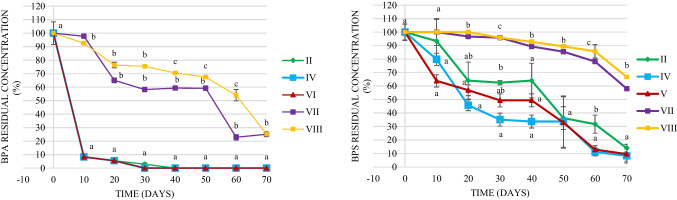
Residual content of BPA and BPS (%) in the studied microcosms. Microcosms: II—water and sediment, BPs, (control); IV—consortium, water, and sediment, BPs; V—consortium, water, and sediment, BPS; VI—consortium, water, and sediment, BPA; VII—consortium, autoclaved water, and sediment, BPs, (control); VIII—consortium, BPs, (control). The data points represent the average of three independent experiments ± standard deviation. Different letter(s) (within each time point) indicate statistical significance (ANOVA followed by Fisher’s LSD test) related to BPs loss in created microcosms at *p*<0.05, based on the effects on microcosms

In this study, the effectiveness of removal of BPs present at a concentration of 10 mg L^−1^ in the created river water and sediment microcosms was examined. Statistical analysis of obtained data showed significant differences (*p*<0.05) both between the time of sampling points and microcosms. Since time has a significant impact on removing pollutants from the environment (Zaborowska et al. 2020), in this study, we analyzed the differences between microcosms at each sampling point. Figure 1 shows the percentage of residual content of BPA and BPS in the non-bioaugmented microcosm (II) and in the microcosms bioaugmented with different treatments (IV–VIII) during a 10-week experiment. As indicated by the data, the removal of BPA proceeded much faster in comparison with BPS removal. BPA disappeared from microcosms II, IV, and VI within 30 days. However, in bioaugmented microcosms without autochthonous bacteria (VII and VIII), BPA was still detected on the last day of the experiment. Regarding BPS, this compound was not completely removed in the tested microcosms during the experimental period. Nevertheless, in bioaugmented microcosms (III, IV, V, and VI), BPS disappearance was significantly higher (*p*<0.05) as compared to microcosms without autochthonous bacteria (VII and VIII). There was no significant difference in BPS removal between bioaugmented microcosms and microcosm II consisting of river water and sediment. The obtained results showed that the introduction of BPs-removing bacteria BG12 and K1MN into the water–sediment microcosms did not significantly increase the removal efficiency of either BPs compared with the non-bioaugmented microcosm II. However, a significant decrease of BPs was observed in bioaugmented microcosms as compared to microcosms VII and VIII, indicating that BPs removal was primarily due to the activity of indigenous microorganisms. The presence of a micture of pollutants may influence the removal of individual substances, which was probably the reason for the low efficiency of BPs removal from these microcosms by the introduced consortium (González-González et al. 2022), especially as BG12 was effective in BPA removal, while K1MN was effective in BPS elimination, but from BSM medium where each of them was a single carbon source (“Bacterial strains and their labeling with a fluorescent protein”). High ability of microorganisms inhabiting water and sediments to removal BPs was observed in previous studies. For instance, Zhou et al. (2020) obsrved that BPA was rapidly removed in a river, suggesting that autochthonous BPA-removing bacteria are widely distributed in the water. The vital role of microorganisms in BPA biodegradation was demonstrated by Tong et al. (2020), who observed the complete inhibition of BPA removal in sterilized sediment contaminated with BPA. Similarly, in microcosms consisting of river water or activated sludges from municipal or village WWTPs, BPS was biodegraded in a range of 40–55% in 52 days (Frankowski et al. 2020). In another study, an activated sludge bioreactor completely removed BPS at a concentration of 50 mg L^−1^ in 10 days (Huang et al. 2019).

Besides the metabolic activity of microorganisms, adsorption is another significant way of BPs removal (Ferrer-Polonio et al. 2021). Therefore, BPs-contaminated microcosms with autoclaved river water and sediment (VII) and BPs-contaminated microcosms composed of BSM and consortium (VIII) were included in the experiment to analyze which of these two strategies contributes to BPs loss in this study. Due to the non-significant differences in BPs content between microcosms VII and VIII, it was concluded that bacterial activity played a key function in BPs removal, which was in accordance with previous results (Sun et al. 2017).

The data presented in Fig. 1 were supported by the values of the removal rate (*k*) and the half-life (*t*_1/2_) constants (Table 2). The obtained *k* and *t*_*1*/2_ values confirmed that removal of BPs was more effective in microcosms II, IV, V, and VI as compared to microcosms VII–VIII, and BPA removal proceeded much faster than BPS removal. Many studies on BPA removal process kinetics have assumed a simple first-order (Rajendran et al. 2017) or even a pseudo-first-order kinetic model (Kohtani et al. 2003). However, Abargues et al. (2018) demonstrated that the second-order kinetic model is suitable for predicting BPA removal, which was confirmed in this study. The second-order kinetic model gave reasonably good fits using a linear least-squares analysis (0.912–0.972). The values *k* and *t*_1/2_ constants showed that the removal rate of BPA decreased in the following order: microcosm II > microcosms IV and VI > microcosms VII and VIII. The BPS removal constants were calculated based on a pseudo-zero kinetic model, which also gave good fits using a linear least-squares analysis (0.919–0.998). The of *k* and *t*_1/2_ constants were the highest for microcosms IV and V followed by microcosm II. Moreover, the constants were at least one order of magnitude smaller than those calculated for BPA. Such values probably result from BPS recalcitrance to microbial removal (Ogata et al. 2013). The first-order kinetic model revealed that BPS was removed faster (*k*= 0.04–0.16 day^−1^, *t*_1/2_= 5.8–17.3 days) during biological wastewater treatment with activated sludge compared to our study (Kovačič et al. 2021). On the other hand, higher persistence of BPS to removal in the surface water of Taihu Lake was observed by Zhou et al. (2020), who studied the removal of eight bisphenols in the natural environment. They found that the amount of BPS at the end of 49-day incubation was almost unchanged in comparison with day 1. The results of our and the studies mentioned above suggest that the efficiency of BPS removal in the natural environment might strongly depend on the structure and richness of microbial communities,

**Table 2:** Rate constant (*k*), values of half-life (*t*_1/2_), and correlation coefficient (*R*^2^) of BPA second-order removal kinetic (A) and BPS pseudo-zero order removal kinetic (B) in the microcosms

Microcosm	*k* (mM/d^−1^)	*t*_1/2_ (d)	*R*^2^
II	0.013	390.625	0.943
IV	0.292	17.105	0.919
V	0.292	17.105	0.919
VII	0.001	5000	0.941
VIII	0.001	5000	0.998

Microcosms: II—water and sediment, BPs, (control); IV—consortium, water, and sediment, BPs; V—consortium, water, and sediment, BPS; VI—consortium, water, and sediment, BPA; VII—consortium, autoclaved water, and sediment, BPs, (control); VIII—consortium, BPs, (control)

### Survival of introduced strains in water–sediment microcosms

**Fig. 2:**
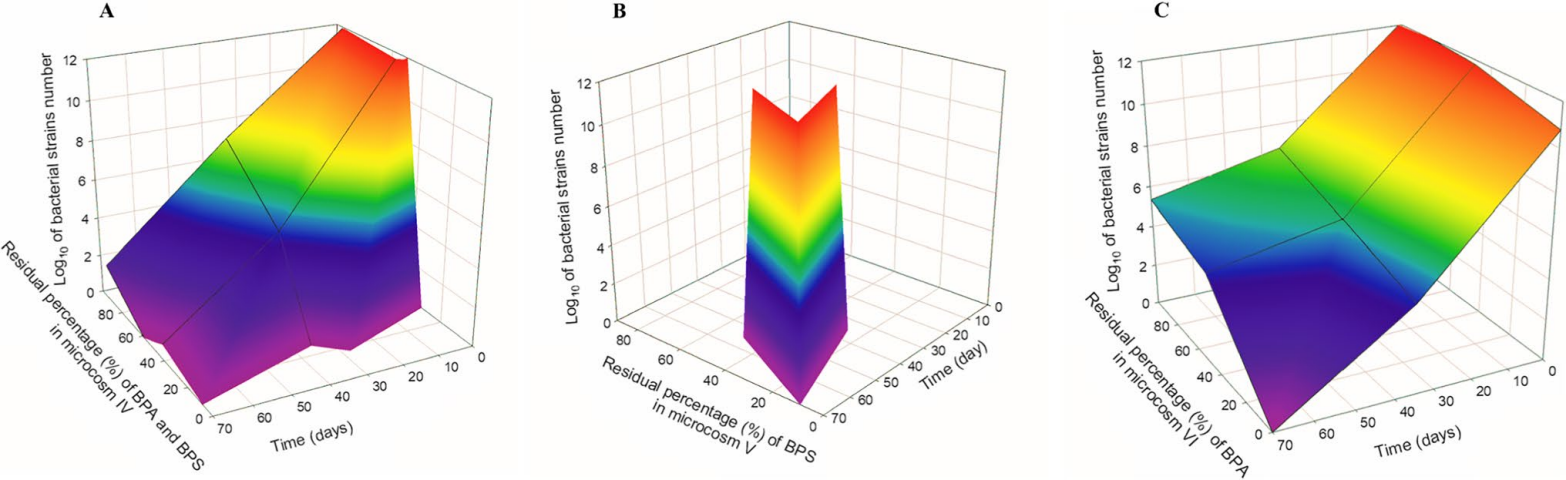
Bacteria number in correspondence to residual concentration of BPA and BPS (**A**), BPS (**B**), or BPA (**C**) and time in microcosms IV, V, and VI. Microcosms: IV—consortium, water, and sediment, BPs; V—consortium, water and sediment, BPS; VI—consortium, water, and sediment, BPA

The effectiveness of bioaugmentation is strongly connected with the survival rate of introduced cells; hence, an important aspect of this study was monitoring the survival of the inoculants. To achieve this goal, BG12 and K1MN were tagged with FP protein and counted under a fluorescent microscope. Using this technique, it was found that neither introduced strain was detected in any microcosms from day 40 (Table S1). The compilation of the results listed in Fig. 1 and Table S1 is presented as a 3D response surface plot in Fig. 2A–C. The red region indicates the region with the highest number of bacteria which includes the sum of both introduced strains, while the blue area represents the region where bacteria were not present. The distribution of the red region was found to be dependent on time and the amount of either BPA or BPS or both of them in microcosms. In microcosm IV (Fig. 2A), it was confirmed that after 35 days of the experiment and below 40% of the initial concentration of both bisphenols, no bacteria were detected. In microcosm V (Fig. 2B), introduced bacteria were not detected at about 50% of the initial BPS concentration and after 35 days of incubation. The number of introduced FP-tagged cells decreased fastest in microcosms amended only with BPA (Fig. 2C). After 20 days of the experiment, bacteria were not detected. So far, only a few studies have focused on monitoring the survival of introduced strains in aquatic environments bioaugmented with selected strains (Zhou and Gough 2016). Such studies allowed us to evaluate whether the higher effectiveness of pollutant removal resulted from the activity of the introduced strains or rather from biostimulation that occurs when dead cells of inoculants serve as an additional source of energy and carbon, leading to increased activity and the abundance of autochthonous microorganisms (Płociniczak et al. 2020). Such an effect was observed after the bioaugmentation of hydrocarbon-contaminated soil with *Rhodococcus erythropolis* CD 106 (Pacwa-Płociniczak et al. 2019). The decline in the number of introduced BG12 and K1MN strains until 30 days and their total lack on the successive sampling days observed in our study might be explained by the loss of planktonic cells due to competing indigenous microbes, protozoan grazing, and suboptimal environmental conditions. Taking these factors into account, the successful application of bioaugmentation is dependent on the use of appropriate bacterial strains featuring resistance to external, abiotic, and biotic factors (Xiong et al. 2017).

### Bacterial community compositions in water-sediment microcosms

To answer the question wheter inoculants and BPs changed the diversity and/or activity of the autochthonous bacterial communities in microniches, these tested microcosms were subjected to high-throughput sequencing of the 16S rRNA gene.

Analysis of samples from treatments I–VI on days 0, 35, and 70 yielded a total of 3,480,480 valid reads. The number of reads varied from 37,104 (sum of reads for sample IV, day 35) to 147,6501 (sum of reads for sample I, day 0) and were grouped into operational taxonomic units (OTUs) (using 97% minimum similarity), and these ranged from 498 (V day 35) to 4615 (I day 0).

**Fig. 3:**
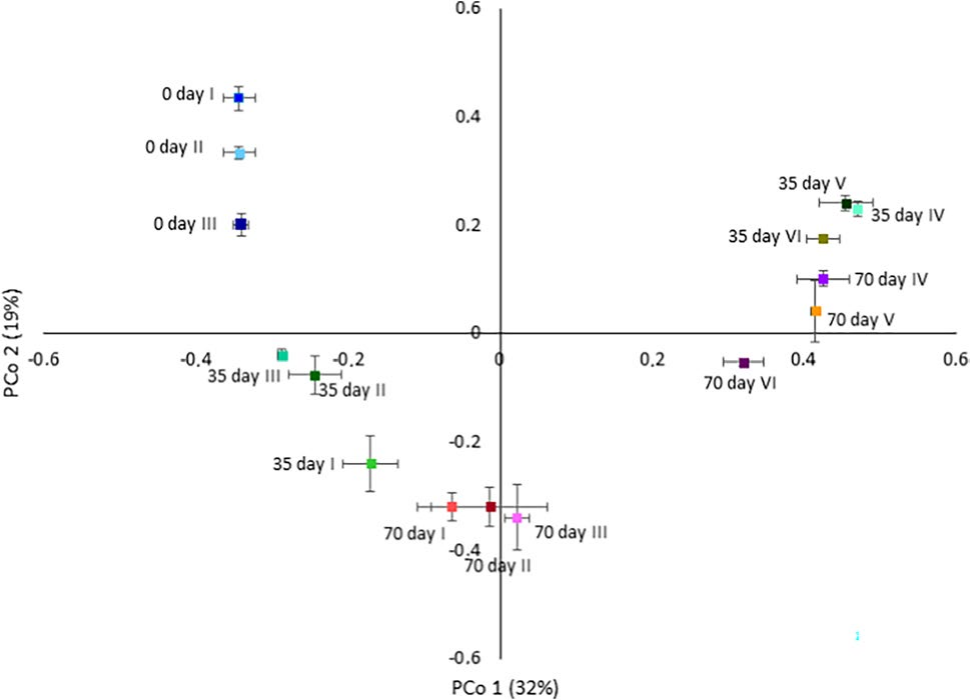
Principle coordination analysis (PcoA) plots based on the Bray-Curtis similarities of the I-based bacterial community analysis of the microcosms. Microcosms: I—water and sediment, (control); II—water and sediment, BPs, (control); III—consortium, water, and sediment, (control); IV—consortium, water, and sediment, BPs; V—consortium, water, and sediment, BPS; VI—consortium, water, and sediment, BPA

High-throughput sequencing analysis and alpha diversity indices are shown in Table S2. Statistically significant (*p* < 0.05) differences in the values of these indices were observed for all tested indices, indicating different species richness between samples from microcosms IV–VI on days 35 and 70. For samples from microcosm IV, significantly different values were obtained for Simpson, for microcosm V for Shannon and Simpson, and for microcosm VI for Chao1 indices. These results were confirmed by principal coordinate analysis (PCoA) based on a *Bray-Curtis dissimilarity matrix* (Fig. 3). For all analyzed microcosms, the microbial diversity was the same at the beginning of the experiment (day 0). Furthermore, clear relative dispersion between microcosms I–III and IV–VI was observed on days 35 and 70, indicating that both bioaugmentation and BPs changed the bacterial community structure. PERMANOVA analysis confirmed the significance of clustering (*p* = 0.001); however, with three replicates for each sample, the clustering was not significant with pair-wise comparisons of the types.

**Fig. 4:**
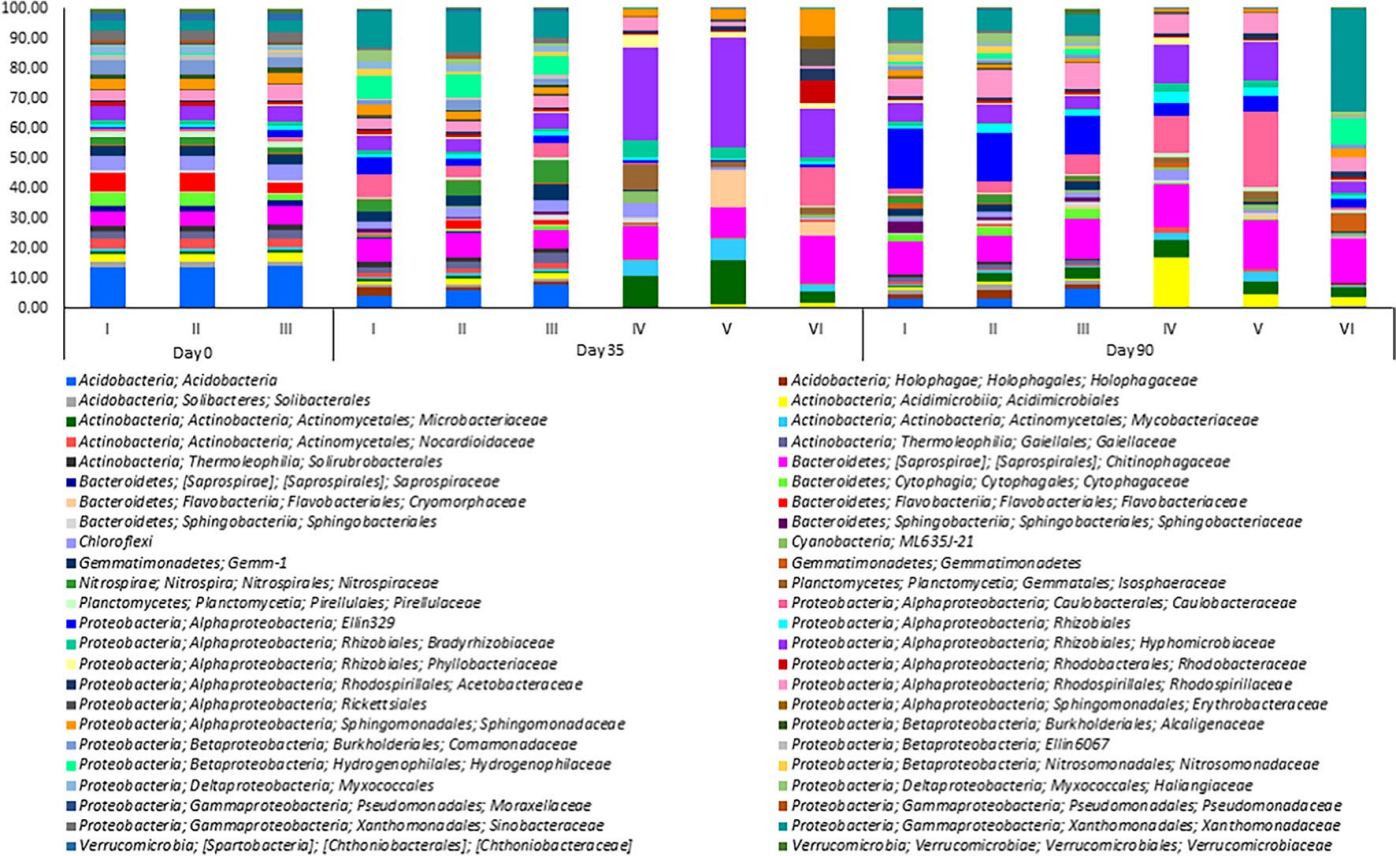
Relative abundance of the bacterial genera in the analyzed microcosms. Microcosms: I—water and sediment, (control); II—water and sediment, BPs, (control); III—consortium, water, and sediment, (control); IV—consortium, water and sediment, BPs; V—consortium, water and sediment, BPS; VI—consortium, water, and sediment, BPA

The taxonomic composition of the bacterial communities in the analyzed microcosms at the phylum level showed the predominance of *Proteobacteria* (including *Alphaproteobacteria*, *Betaproteobacteria Gammaproteobacteria*, and *Deltaproteobacteria*) *Acidobacteria*, *Actinobacteria*, and *Bacteroidetes* were abundant in all the samples at the beginning of the experiment (Fig. 4). This finding is in accordance with outcomes reported previously, showing that *Proteobacteria* members are responsible for the removal of diverse phenolic compounds, including BPA (Czarny et al. 2020). Cydzik-Kwiatkowska et al. (2017) revealed that the abundances of *Proteobacteria* and *Bacteroidetes* were over 0.5% of all identified sequences, which was the highest percentage of bacteria in aerobic granules used to treat wastewater containing BPA in a concentration up to 12 mg L^−1^. This suggests that the main controlling factor affecting the relative abundance of the abovementioned phyla is likely to be the concentration of bisphenols in the treated microcosms.

During the removal, a remarkable difference in the bacterial community between microcosms I–III and microcosms IV–VI was found. In those first groups of samples, the bacterial community was comparable and composed primarily of four different phylogenetic groups at the family taxonomic rank: *Xanthomonadaceae*, *Hydrogenophilaceae*, *Chitinophagaceae*, and *Nitrospiraceae*. In microcosms IV–VI, the most dominant bacterial families in all samples were *Hyphomicrobiaceae* (including the genus *Hyphomicrobium* with an abundance of 12.88% for microcosm IV, 13.64% for microcosm V, and 6.66% for microcosm VI; data not presented), *Chitinophagaceae*, and *Microbacteriaceae*. Additionally, the *Isosphaeraceae* family was also present in high abundance in microcosms IV, *Cryomorphaceae* in microcosm V, and *Caulobacteraceae* in microcosm VI. No reads belonging to the families *Pseudomonadaceae* and *Moraxellaceae* were detected.

On day 70, the re-organization of the bacterial community compared to days 0 and 35 was observed. Bacterial community structures in microcosms I–III were comparable and composed mainly of *Ellin 329*, *Chitinophagaceae*, *Xanthomonadaceae*, and *Rhodospirillaceae.* In microcosm IV *Acidimicrobiales* followed by *Chitinophagaceae*, *Caulobacteraceae*, and *Hyphomicrobiaceae* comprised the main dominant group. These two latter families also dominated in microcosm V, while *Xanthomonadales* followed by *Chitinophagaceae* dominated in microcosm VI.

**Fig. 5:**
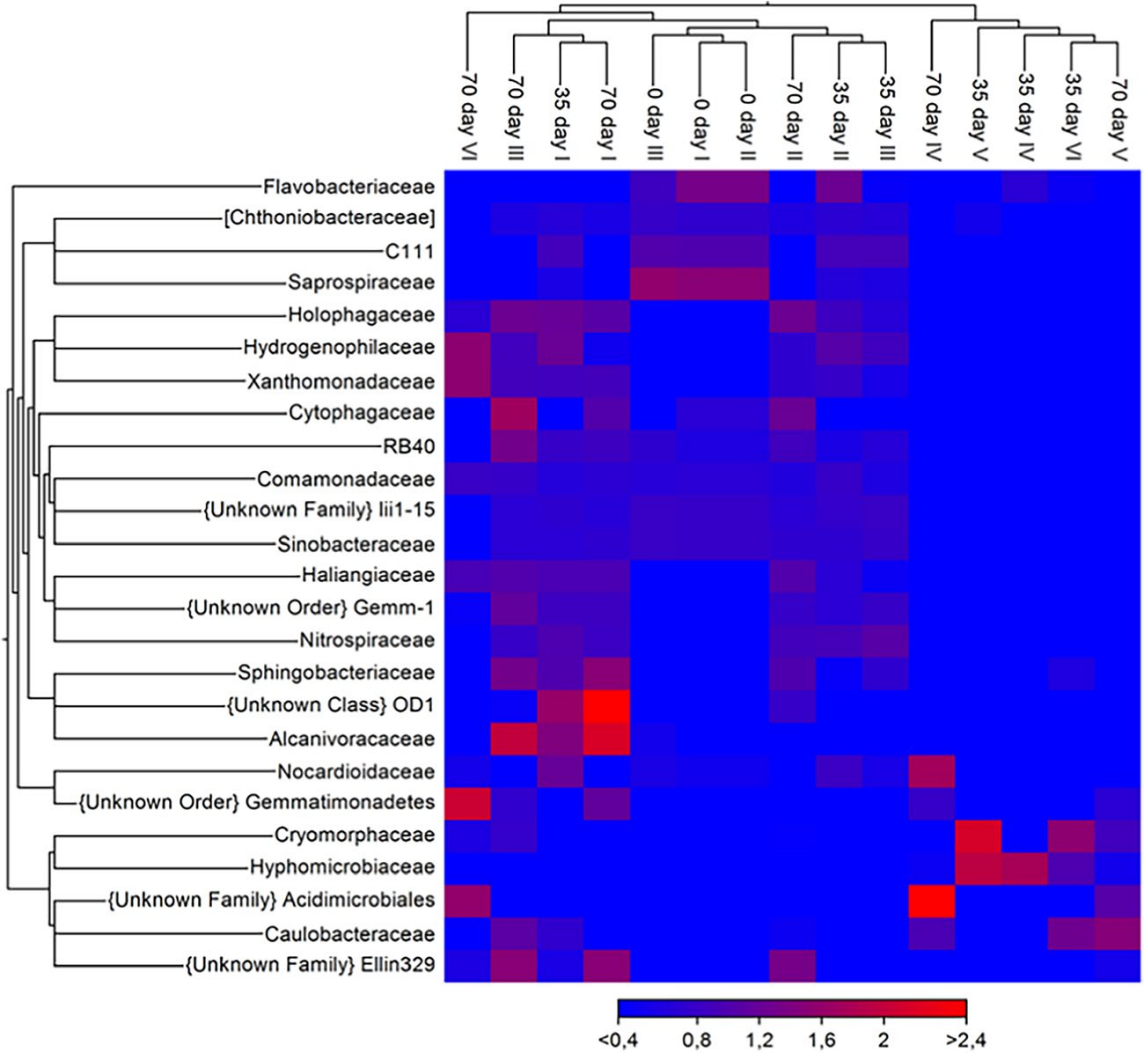
Heat map of relative abundance of OTUs with the highest abundance. Samples and taxa were clustered using the Euclidean distance and hierarchical clustering with the average linkage method. Microcosms: I—water and sediment, (control); II—water and sediment, BPs, (control); III—consortium, water, and sediment, (control); IV—consortium, water, and sediment, BPs; V—consortium, water, and sediment, BPS; VI—consortium, water, and sediment, BPA

To better understand the BPs removal process, a heat map clustering analysis of the top 18 most abundant taxa in the tested microcosms was used (Fig. 5). It illustrates how the bacterial population changed depending on the duration of the incubation period and the type of BPs analog. Comparison of the microbial community composition of microcosms from different treatments and times of the experiment performed using the OTUs with the highest abundance distinguished two clusters. The first cluster included microcosms I–III of all tested days, and microcosm VI of day 70, while the second cluster included microcosms IV and V of days 35 and 70 and microcosm VI of day 35. As Fig. 4 shows, during the removal, the proportions of *Acidimicrobiales* and *Caulobacteraceae* in microcosms IV and V on day 70 exceeded *Hyphomicrobiaceae* and *Cryomorphaceae*, which were dominant on day 35. In bioaugmented microcosm VI amended only with BPA, *Cryomorphaceae*, *Hyphomicrobiaceae*, and *Caulobacteraceae* were prevalent on day 35.

These basic findings are consistent with research showing that members of *Bacteroidetes*, *Chloroflexi*, *Gemmatimonadetes*, *Actinobacteria*, and *Planctomycetes* were the major bacterial groups in BPA-removing anaerobic sediments (Yang et al. 2015). In another study *Cyanobacteria*, *Chloroflexi*, *Planctomycetes*, and *Acidobacteria* were the second predominant phyla present in mangrove sediment contaminated with endocrine-disrupting chemicals, including BPA (Yuan et al. 2017). *Hyphomicrobiaceae*, especially *Hyphomicrobium*, *Caulobacteraceae*, *Chitinophagaceae*, and *Cryomorphaceae* were previously found to metabolize different hardly degradable xenobiotics (Wang et al. 2019). This may explain their high abundance in microcosms IV–VI.

**Fig. 6:**
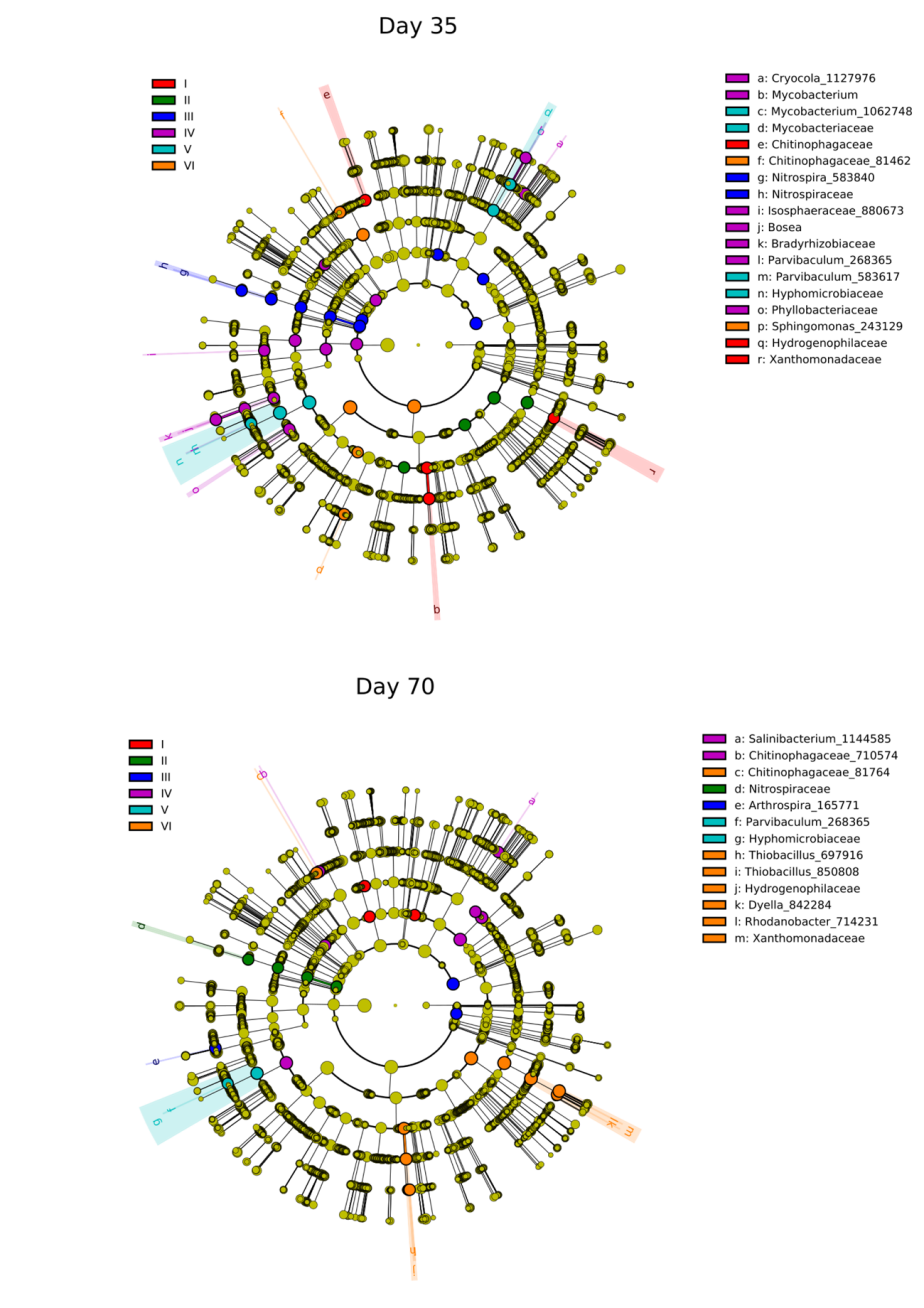
The taxonomic cladograms of the most differentially abundant bacterial taxa obtained by LEfSe analysis of 16S sequences on days 35 and 70. Microcosms: I—water and sediment, (control); II—water and sediment, BPs, (control); III—consortium, water, and sediment, (control); IV—consortium, water, and sediment, BPs; V—consortium, water, and sediment, BPS; VI—consortium, water, and sediment, BPA

To further identify the microbial biomarkers in each treatment, a LEfSe analysis was applied. Figure 6 shows the bacterial groups among each of the six microcosms on days 35 and 70 with significant differences with log10 (linear discriminant analysis [LDA] scores) > 4.0, while Figure S1 shows those with log10 (LDA scores) > 2.5. No significant differences for tested microcosms on day 0 were obtained. LEfSe analysis revealed that different taxa were significantly enriched in the different treatments, thus indicating that the various niches in different microcosms could select for specific microbial community members. Bacteria taxa that differ significantly in microcosms II–VI, besides *Chitinophagaceae*, were not considered core community members in microcosm I composed only of river water and sediment. Remarkably, in the same microcosms sampled on different days, significantly high abundance of various bacterial taxa was detected. Only the *Hyphomicrobiaceae* family and *Parvibaculum* genus were present in significantly high abundance in microcosms V on both tested days. Interestingly, the *Sphingomonas* genus was enriched considerably in microcosm VI on day 35. Therefore, we speculate that these bacteria could use BPS as a carbon substrate. Moreover, high abundance of *Sphingomonas* sp. was found in microcosm VI amended only with BPA. Previously, *Sphingomonas* strains were demonstrated to be good BPA-degraders (Spivacks et al. 1994).

To sum up, no reads belonging to *Pseudomonas* and *Acinetobacter* in all microcosms on days 35 and 70 were found. This confirms that the introduced bacteria did not survive in the microcosms after day 30 of the experiment. Moreover, the composition of the active BPs-removing communities during the 10-week incubation period was shaped by the bioaugmentation and the addition of BPs and was altered to achieve accelerated BPs removal. From this standpoint, reconstruction of the bacterial community may be due to the selection and maintenance of BPs-removing strains in treated microcosms until BPs have been converted and/or the appearance of strains that utilize intermediates of BPs removal. These results revealed that both bioaugmentation and BPs changed the composition of major bacterial groups. Most of the dominant bacterial groups which were present on day 35 can remove BPA, while those present during the whole incubation period can degrade/transform BPS. These findings agree well with previous studies in which the microbial community has also been shifted during the removal of various organic compounds (Huang et al. 2019).

### Predicted metabolic functions of bacterial communities in BPs-amended microcosms

To complete the information about genes responsible for metabolic pathways, a more detailed analysis of differences in genes and pathways abundances potential between the microcosms without addition of BPs (I, III) and BPs-amended microcosms (II, IV, V, VI) was performed. The functional prediction of the metagenome of the microcosms from days 35 and 70 revealed the presence of 432 pathways and 2339 proteins. PICRUSt2 detected in the analyzed microcosms pathways and proteins, which were annotated to the following categories: genetic information processing, metabolism, cellular processes, and environmental information processing. The most significant differences were detected in the abundance of genes of the protocatechuate removal pathway, which were predominant on day 35 in microcosm II and day 70 in treatments II and IV (P23-PWY; Figure S2), thus suggesting that autochthonous microflora may utilize BPs as carbon sources. Previously, it was found that BPA and BPS are removed to many intermediates, including catechol (Ogata et al. 2013). Those results, as well as the present findings are in line with a previous analysis demonstrating that protocatechuate removal might be involved in BPA transformation (Zhou et al. 2015).

In addition, Cydzik-Kwiatkowska et al. (2021) found high abundance of genes coding enzymes responsible for benzoate removal in BPA-exposed aerobic granular sludge. This is in accordance with the present results, as 4-carboxymuconolactone decarboxylase (EC.4.1.144) was the most abundant protein in BPA-amended microcosms. This may be related to the structure of BPA, which contains acetate. Moreover, high abundance of glutathione transferase (EC.2.5.1.18) engaged in the metabolism of xenobiotics by cytochrome P450 was observed in microcosms amended with BPs. This enzyme has been demonstrated to be responsible for the removal of a large variety of compounds, including chlorinated biphenyls and creosote (Brennan et al. 2009; Smułek et al. 2020).

## Conclusion

In this study, we analyzed the loss of BPs at a concentration of 10 mg L^−1^ in water–sediment microcosms bioaugmented with the bacterial consortium. The results showed that BPA in microcosms II, IV, and VI was eliminated within 30 days. In contrast, BPS in microcosms II, IV, and V was still detected on day 70; however, its amount significantly decreased over the experimental period. The introduced consortium survived in tested treatments for up to half the duration of the experiment. These results support the view that degradation by indigenous microflora plays a key role in removing BPs. Moreover, the processes occurring during BPs transformation modulated the structure of the autochthonous bacterial communities. The dominance of *Cryomorphaceae*, *Hyphomicrobiaceae*, and *Caulobacteraceae* families in BPs treated microcosms indicates their role in removal of these pollutants. Bioaugmentation remodeled the bacterial community as well; however, it did not have any impact on the removal of BPs in the microcosms. Therefore, understanding the issues related to removal of BPs from aquatic systems requires further detailed research including monitoring of bacterial, functional expression of key enzymes in BPs degradation as well as the effect of BPs intermediates on the bacterial community. The next stage of this research will focus on clarifying these issues.

## Supplementary Information

ESM 1 (DOCX 27 kb)

## Data Availability

All data generated or analyzed during this study are included in this published article.
