# Supplementary material for: The microbial removal of bisphenols in aquatic microcosms and associated alteration in bacterial community
Source: Environ Sci Pollut Res Int. 2023 Jun 29;30(36):85292–304. doi: 10.1007/s11356-023-28305-2 (PMC10404205; doi:10.1007/s11356-023-28305-2)
Supplement: Supplementary file 1 — (DOCX 27 kb) [file 11356_2023_28305_MOESM1_ESM.docx]

**The microbial degradation of bisphenols in bioaugmented and non-bioaugmented river water-sediment microcosms**

Magdalena Noszczyńska^a*^, Magdalena Pacwa-Płociniczak^a^, Kinga Bondarczuk^b^, Zofia Piotrowska-Seget^a^,

^a^ Institute of Biology, Biotechnology and Environmental Protection, Faculty of Natural Sciences, University of Silesia in Katowice, Jagiellońska 28, 40-032 Katowice, Poland

^b^ Centre for Bioinformatics and Data Analysis, Medical University of Bialystok, Białystok, Poland

*Corresponding author: magdalena.noszczynska@us.edu.pl; 0048 32 2009 442; ORCID iD: 0000-0002-3558-7409

Figure S1 is provided as a pdf file.

**Figure S1.** Linear discriminant analysis (LDA) scores. Biomarkers are shown that differ significantly (LDA > 2.5) in the six microcosms on day 35. Red colour indicates microcosm I; green – microcosm II; blue – microcosm III; purple – microcosm IV; turquoise – microcosm V; light green – microcosms VI. Microcosms: IV - consortium, water and sediment, BPs; V - consortium, water and sediment, BPS; VI - consortium, water and sediment, BPA.

Figure S2 is provided as a pdf file.

**Figure S2.** Linear discriminant analysis (LDA) scores. Biomarkers are shown that differ significantly (LDA > 2.5) in the six microcosms on day 70. Red colour indicates microcosm I; green – microcosm II; blue – microcosm III; purple – microcosm IV; turquoise – microcosm V; light green – microcosms VI. Microcosms: IV - consortium, water and sediment, BPs; V - consortium, water and sediment, BPS; VI - consortium, water and sediment, BPA.

**Table S1**. The number of *Pseudomonas* sp. BG12 and *Acinetobacter* sp. K1MN strains in the microcosms.

| **Time (days)** | **Microcosm** | **Log_10_ number of bacteria ( Log_10_** | |
| --- | --- | --- | --- |
|  |  | **BG12** | **K1MN** |
| 0 | IV | 6.08±0.52 ^a^ | 6.02±0.52  ^a^ |
|  | V | 6.07±0.54  ^a^ | 6.05±0.57  ^a^ |
|  | VI | 6.04±0.85 ^a^ | 6.11±0.42  ^a^ |
| 10 | IV | 5.08±0.41  ^a^ | 5.68±1.21  ^a^ |
|  | V | 4.83±0.98  ^b^ | 5.56±0.13  ^a^ |
|  | VI | 4.83±0.36  ^b^ | 4.57±0.35  ^b^ |
| 20 | IV | 5.27±0.83  ^a^ | 4.82±0.42  ^ab^ |
|  | V | 5.02±0.54  ^a^ | 5.29±0.63  ^a^ |
|  | VI | 4.55±0.44  ^b^ | 4.60±0  ^b^ |
| 30 | IV | 4.02±0.85  ^a^ | 4.30±0  ^a^ |
|  | V | 0 ^b^ | 4.08±0.66  ^a^ |
|  | VI | 0 ^b^ | 0 ^b^ |

The data points represent the average of ten independent experiments ± standard deviation. Different letter(s) (within each day) indicate statistical significance (ANOVA followed by Fisher’s LSD test) related to bacterial counts in created microcosms at *p<0.05*, based on the effects on microcosms. Microcosms: IV - consortium, water and sediment, BPs; V - consortium, water and sediment, BPS; VI - consortium, water and sediment, BPA.

**Table S2.** High-throughput sequencing analysis and alpha diversity indices of the analyzed treatments.

| **Day** | **Treatment** | **OTUs** | **Chao1** | **Shannon** | **Simpson** |
| --- | --- | --- | --- | --- | --- |
| 35 | IV | 622 ± 51^a^ | 717 ± 11^a^ | 5.51 ± 0.27^a^ | 0.94 ± 0.00^a^ |
|  | V | 573 ± 80^a^ | 682 ± 113^a^ | 4.67 ± 0.25^a^ | 0.90 ± 0.02^a^ |
|  | VI | 737 ± 18^a^ | 854 ± 21^a^ | 5.73 ± 0.29^a^ | 0.94 ± 0.02^a^ |
| 70 | IV | 686 ± 14^a^ | 791 ± 14^a^ | 6.32 ± 0.08^b^ | 0.97 ± 0.00^a^ |
|  | V | 758 ± 112^a^ | 890 ± 109^a^ | 6.44 ± 0.50^b^ | 0.95 ± 0.03^a^ |
|  | VI | 673 ± 97^a^ | 840 ± 108^a^ | 5.93 ± 0.41^b^ | 0.95 ± 0.03^a^ |
| MSV | | - | - | Time |  |

± Stand. dev. of three independent experiments. Different letters (within each group) indicate significant differences (*p < 0.05*, LSD test), considering the effects of the time, MSV – major source of variance.
